# Pathway reporter genes define molecular phenotypes of human cells

**DOI:** 10.1186/s12864-015-1532-2

**Published:** 2015-04-24

**Authors:** Jitao David Zhang, Erich Küng, Franziska Boess, Ulrich Certa, Martin Ebeling

**Affiliations:** Pharmaceutical Research and Early Development, Pharmaceutical Sciences, Roche Innovation Center Basel, F. Hoffmann-La Roche AG, Grenzacherstrasse 124, 4070 Basel, Switzerland

**Keywords:** Molecular phenotype, Pathway analysis, Gene expression, Pathway reporter genes

## Abstract

**Background:**

The phenotype of a living cell is determined by its pattern of active signaling networks, giving rise to a “molecular phenotype” associated with differential gene expression. Digital amplicon based RNA quantification by sequencing is a useful technology for molecular phenotyping as a novel tool to characterize the state of biological systems.

**Results:**

We show here that the activity of signaling networks can be assessed based on a set of established key regulators and expression targets rather than the entire transcriptome. We compiled a panel of 917 human *pathway reporter genes*, representing 154 human signaling and metabolic networks for integrated knowledge- and data-driven understanding of biological processes. The reporter genes are significantly enriched for regulators and effectors covering a wide range of biological processes, and faithfully capture gene-level and pathway-level changes. We apply the approach to iPSC derived cardiomyocytes and primary human hepatocytes to describe changes in molecular phenotype during development or drug response. The reporter genes deliver an accurate pathway-centric view of the biological system under study, and identify known and novel modulation of signaling networks consistent with literature or experimental data.

**Conclusions:**

A panel of 917 pathway reporter genes is sufficient to describe changes in the molecular phenotype defined by 154 signaling cascades in various human cell types. AmpliSeq-RNA based digital transcript imaging enables simultaneous monitoring of the entire pathway reporter gene panel in up to 150 samples. We propose molecular phenotyping as a useful approach to understand diseases and drug action at the network level.

**Electronic supplementary material:**

The online version of this article (doi:10.1186/s12864-015-1532-2) contains supplementary material, which is available to authorized users.

## Background

An important goal of gene expression analysis is to capture pathway-level changes of the biological system in response to external stimuli or environmental changes [1]. For mammalian cells, the major signaling and metabolic pathways have been charted [2-4]. While many of the primary events in signaling networks consist of protein-level interactions and modifications, the essential response to a stimulus is at the transcriptional level. While the precise transcriptional consequences of activating a given pathway strongly depend on the biological context (such as cell type, and cross-talk with other pathways), knowledge of existing pathways and surveys of large-scale expression experiments suggest the possibility that knowledge of the expression levels of selected genes may be sufficient to infer activation states of most described pathways in a broad range of different biological contexts [5].

In the early days of functional genomics, pathway activity was studied by applying quantitative reverse transcription polymerase chain reaction (qRT-PCR) to manually curated transcriptional targets of pathways, and/or performing reporter assays designed for selected transcription factors. With the advent of high-throughput gene expression profiling platforms such as microarrays and RNA-sequencing, transcriptional research focuses on whole-transcriptome analysis.

Technical constraints of the high-throughput platforms including microarray and quantitative RNA sequencing impact the accurate estimation of transcript abundances and consequently differential gene expression. Background noise [6] and other factors such as hybridization artifacts introduce significant systematic error in any of the current microarray platforms [7]. Precise quantification by conventional RNA-sequencing is complicated by the difficulty of multiple read mapping among homologs. In addition, low expressed genes are only poorly covered when the whole transcriptome is subject of a sequencing experiment [8]. In drug research and development, it is desirable to monitor multiple pathways in cells of different origins to study both efficacy and adverse effects at the same time. A multiplex assay allowing systematic understanding of pathway-level responses that matches industrial screening capacities is needed. A better understanding of diseases at pathway levels and subsequent pathway-guided drug development may reduce the high attrition rates during the drug discovery process [9].

In the current study, we introduce the novel concept of a “molecular phenotype” defined by the activation states of signaling and metabolic pathways. The genes needed to monitor a given pathway we term “pathway reporter genes”. Here, we use a panel of 917 reporter genes compiled based on information from public and proprietary databases covering about 150 human metabolic and signaling networks. We show that molecular phenotyping allows fast and accurate pathway delineation in complex biological systems.

## Results

### Selection of pathway reporter genes

**Figure 1:**
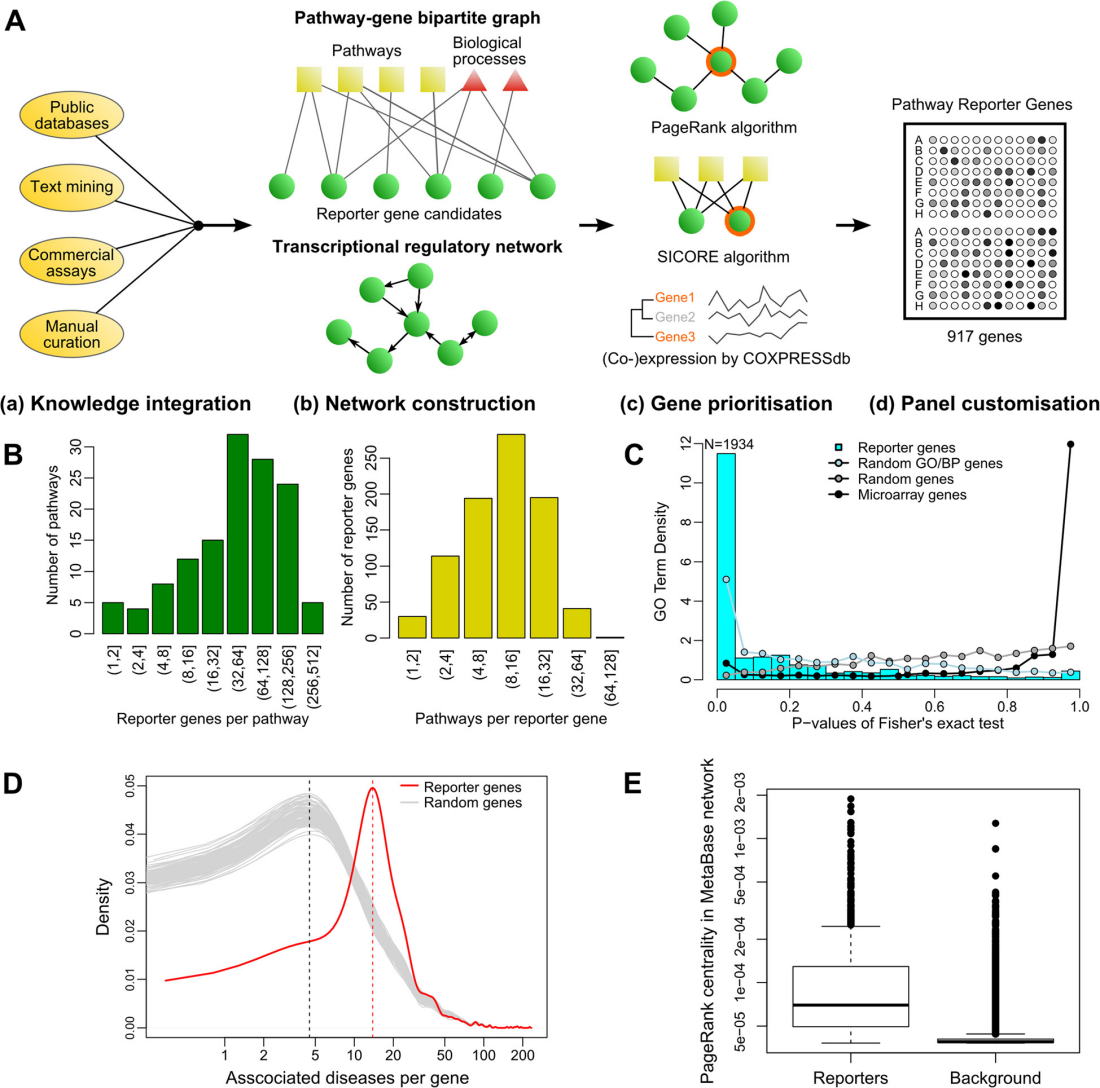
Selection, characterization, and *in silico* validation of pathway reporter genes. **(A)** The selection workflow. Eclipses indicate data sources, green dots candidate genes, boxes the selected biological pathways, and triangles the selected biological processes. The pathway-gene association graph is bipartite and non-directional, while the transcriptional regulatory network consists exclusively of genes and is directional. All steps but panel customization is performed using the internal data infrastructure and software pipeline. **(B)** Histograms of reporter gene counts per pathway, and pathways per reporter gene, respectively. **(C)** Functional enrichment analysis results of reporter genes, randomly selected genes with high-confidence GO/BP annotations, randomly selected genes irrespective of annotations, and genes represent on a typical microarray (Illumina HT-6), respectively. Counts of GO BP terms falling in each Benjamini-Hochberg adjusted *p-*value bin are represented by bars (in case of reporter genes) or dots. The leftmost bin corresponding to adjusted *p* < 0.05: 1934 GO terms are significantly overrepresented by the pathway reporter genes using this threshold. **(D)** Density plot of number of diseases associated with differential expression of pathway reporter genes (red line), compared with associations with randomly selected genes (gray lines, repeated N = 100). **(E)** PageRank centrality of pathway reporter genes in the MetaBase transcriptional regulatory network compared with that of other human genes (*background*).

Pathway reporter genes were selected by a software pipeline integrating various data sources in four steps: knowledge harmonization, information integration, gene prioritization, and panel design (Figure 1A).

The first step associates pathways and genes based on causal relationships, i.e. when mRNA or protein expression is associated with the pathway’s activity, either as a key regulator or as an expression target of the pathway. Before the harmonization step, we prioritized 154 signaling pathways, metabolic pathways, and biological processes (abbreviated as ‘pathways’ hereafter) that are of interest to our current drug discovery programs (Additional file 1). Pathway-gene associations were then imported from various sources: (1) public databases including REACTOME [2], PID/NCI-Nature [3], and String DB [10]; (2) text mining results of MEDLINE-indexed abstracts; (3) commercial PCR panels provided by QIAGEN [11] and Bio-Rad [12]; (4) manual curation of articles and reviews published in peer-reviewed journals. Relationships were deliberately not confined in any way to special cell types, tissues, or biological contexts.

Next we constructed a bipartite network between genes and pathways where edges exclusively link gene nodes to pathway nodes, and a transcriptional regulatory network consisting of genes only. Network analysis was used to prioritize reporter genes that are representative for selected pathways. First we ranked the genes using the PageRank centrality [13] in the underlying unidirectional backbone of the transcriptional regulatory network, which assigns higher importance to genes that are attached to other important genes. This centrality measure derives from the eigenvector centrality and has been successfully applied among others in disease diagnostic marker prioritization [14]. Next, we applied two consecutive filters to the gene list: one based on our previously developed SICORE algorithm which identifies gene pairs that share similar pathways as judged by the bipartite graph [15], and the other based on co-expression patterns in large-scale expression profiling experiments stored in COXPRESSdb [16]. Both filters de-prioritize genes of lower centrality that share redundant information with genes of higher centrality.

Finally, we chose the top twenty percent of non-redundant genes ranked by the PageRank centrality, because they cover almost all upstream transcriptional regulators we collected in the knowledge harmonization step and they fit the capacity of AmpliSeq panels (<1,200 genes reported by the vendor). Subsequently these genes were submitted to the IonTorrent web interface for amplicon-specific primer design. Eight genes were removed from the panel following vendor’s recommendations because they are highly expressed in many tissues, thereby reducing the dynamic range of the assay. The panel in its current form consists of 917 genes (identifiers and sequences of designed primers can be found in Additional file 2). Most genes are associated with more than one pathway, and *vice versa* (Figure 1B). By probing expression of pathway reporter genes, we gain a multiplex view on the activation patterns of pathways that are involved in multiple biological processes of interest.

### *In silico* validation of pathway reporter genes

The pathway reporter genes were selected from key regulators and expression targets of various biological pathways to ensure that they are engaged in a wide range of biological processes. To test this, we used Biological Process (BP) terms from Gene Ontology (GO) [17], and tested whether the reporter genes are enriched for effectors of biological processes (Figure 1C). 1934 terms were significantly over-represented in the selection of pathway reporter genes (Fisher’s exact test, Benjamini-Hochberg adjusted *p* < 0.05), ten-fold more than for a list of randomly selected genes and two-fold more than for a list of randomly selected genes with GO annotations of high confidence. An internal benchmark further showed that the enrichment is 1.8-fold higher than the enrichment of a gene list derived from a data-driven feature selection strategy using principal component analysis.

For applications such as disease understanding and drug characterization, it is expected that the selected genes and associated pathways are disease-relevant, i.e. the reporter genes are associated with more diseases than a randomly selected gene set. To test this, we identified diseases that are associated with pathway reporter genes, and compared them to diseases that are associated with 1,000 sets of the same number of randomly selected genes using the gene-disease association database DisGeNet [18] (Figure 1D). Indeed, on average the pathway reporter genes are associated with three times as many diseases as an equal number of randomly selected genes (*p* < 2E-16, Kolmogorov-Smirnov test).

Finally, we crosschecked the quality of gene selection using MetaBase, a proprietary database that annotates gene regulatory networks by manual curation of full-text literature and was not used for network construction [19]. We compared PageRank centrality of pathway reporter genes in the MetaBase network to that of other genes, and found that the median of the former is almost two times higher than that of the latter (8.0E-4 and 2.3E-4, respectively), and many top-ranking genes of the MetaBase network are already included (Figure 1E). This implies that the pathway reporter genes are likely to be more relevant in transcriptional regulatory networks than other genes, and that the PageRank algorithm delivers robust gene prioritization using transcriptional networks curated by different processes.

Examining all gene-gene interactions defined by either direct or indirect transcriptional regulation recorded in MetaBase, we found that 567 of our 917 selected pathway reporter genes act as upstream regulators in 46%, and as downstream regulatory targets in 15% of all the interactions. Taken together, the selected reporters, though comprising only about 4% of the human transcriptome, are involved in more than half (53%) of the annotated gene-gene interactions. In contrast, a list of 917 randomly selected genes are involved on average in 8% interactions (95% confidence interval 5%-13%, bootstrapping N = 500). Therefore, by profiling pathway reporter genes’ expression and leveraging the network structure, it is possible to infer both upstream and downstream gene expression and pathway activity patterns using established methods, e.g. reverse causal reasoning [20].

### Molecular phenotyping of cardiomyocyte development

Both epidemiological and clinical studies suggest the existence of a diabetic cardiomyopathy in humans. The underlying pathogenesis is however only partially understood [21] and there are currently no *in vitro* models that capture both genetic and environmental factors of the disease. As a first step to address this question, we applied molecular phenotyping to human induced pluripotent stem-cell (iPSC) derived cardiomyocytes at day 0, 10, 20, and 60 of the differentiation protocol to query pathway-level changes. In addition, we applied commercially available microarrays to measure the whole transcriptome at the same time points. Previously we have reported a good correlation of gene expression levels determined by the two platforms [22]. In this study we focus on global expression patterns and pathway-level analysis based on molecular phenotyping.

**Figure 2:**
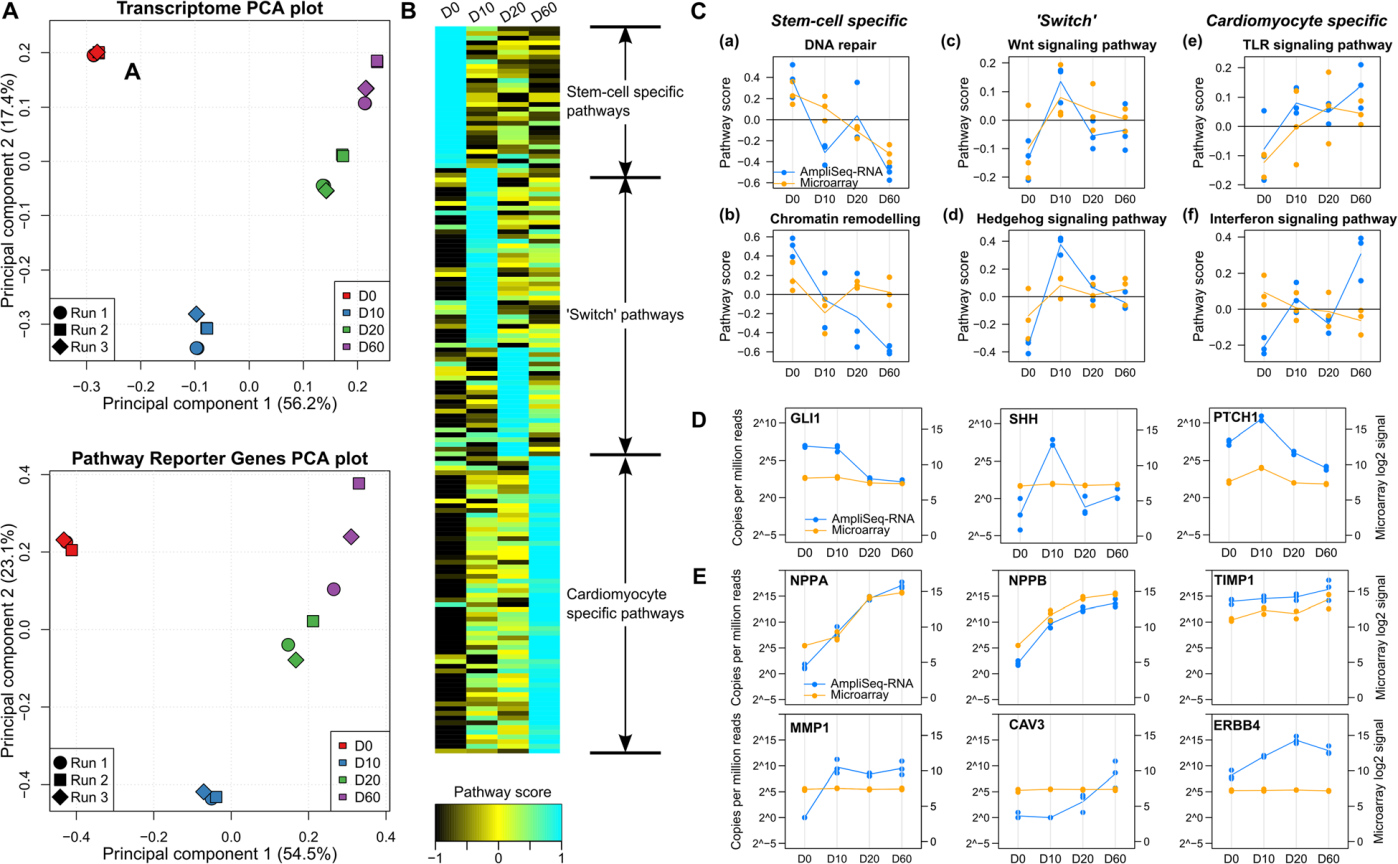
Molecular phenotyping of iPSC-derived cardiomyocyte differentiation. **(A)** Principal component analysis using microarray data of ~22,000 genes (top) and AmpliSeq-RNA data of 917 pathway reporter genes (bottom). **(B)** Pathway activity scores based on expression of pathway reporter genes reported by the GSVA algorithm: pathways are sorted by the time when its activity score reaches the maximum. GSVA scores were calculated for each sample and the average values of each pathway at each time point are reported. To allow between-pathway comparisons, the scores are normalized by row using the z-score transformation (zero mean and unit variance). Pathway names can be found in Additional file 3. **(C)** Activity patterns of selected pathways. Two representatives are chosen from stem-cell specific, ‘switch’, and cardiomyocyte-specific pathways, respectively. **(D)** Expression of key reporter genes of the Hedgehog pathway. **(E)** Expression of selected functional markers of pathways associated with differentiated cardiomyocytes. For comparison, subfigure c, d, and e also include the activity scores and expression profiles reported by microarray.

First, we reveal global gene expression patterns during differentiation with principal component analysis (PCA), using expression of either all genes, or pathway reporter genes alone (Figure 2A). PCA transforms gene expression values into orthogonal features (i.e. principal components) so that each component aligns with the direction of the maximal variance that remains unexplained by previous components. Microarray data and molecular phenotyping data showed almost identical patterns of sample clustering, suggesting that pathway reporter genes (N = 917) capture between-sample variability almost as well as the whole transcriptome (N > 20,000). Although a set of ~1,000 randomly selected genes could also capture the between-sample variability ([23] and simulation), simulation showed that the pathway reporter genes showed significantly larger total variance than randomly selected genes even if they are both profiled with microarrays: 5.5% of total variance of all genes are explained by the reporter genes, versus an average of 1.9% (95% confidence interval: 1.6%-2.5%) by randomly selected genes.

To build a pathway-centric overview of cardiomyocyte differentiation, we transformed pathway reporter genes’ expression into pathway activity scores with the gene set variation analysis (GSVA) method [24]. Molecular phenotyping revealed three types of pathways with distinct temporal patterns (Figure 2B and Additional file 3) stem-cell specific pathways, ‘switch’ pathways whose activities are transiently induced, and cardiomyocyte-specific pathways. Stem-cell specific pathways include key biological processes that are important for stemness maintenance, such as DNA repair and chromatin remodeling. Switch pathways are enriched for signaling pathways that are important for differentiation and cell survival, such as Wnt signaling pathway, Hedgehog signaling pathway, fatty acid metabolism, and PI3K/AKT pathway. Most cardiomyocyte-specific pathways are associated with cell proliferation and cell-type specific functions, such as EGF/PDGF pathway, calcineurin/NF-AT signaling pathway, glutamate signaling pathway, insulin signaling pathway, glucocorticoids signaling pathway, and a set of immune-response pathways such as interferon response pathway and Toll-like receptor (TLR) pathway.

Pathway activation patterns identified by molecular phenotyping provide unprecedented insights into the differentiation of human iPSC-induced cardiomyocytes. For example, it has been long observed that terminally differentiated human neurons display attenuated global DNA repair genes [25], which is observed also in several other cell types (reviewed in [26]). Our data suggest this also applies to cardiomyocytes (Figure 2C (a)). Similarly, we observed gradual repression of the chromatin remodeling pathway (Figure 2C (b)), which is essential for stem-cell self-renewal and inactivation of which is associated with differentiation [27].

Lian *et al.* [28] reported that temporal modulation of Wnt signaling is both essential and sufficient for efficient cardiac function under defined, growth factor-free conditions. We observed a strong activation of Wnt pathway at day 10 which then decreased (Figure 2C (c), also see Figure 3 of [22]), suggesting the pathway’s role in cardiomyocyte development is transient and time-dependent.

**Figure 3:**
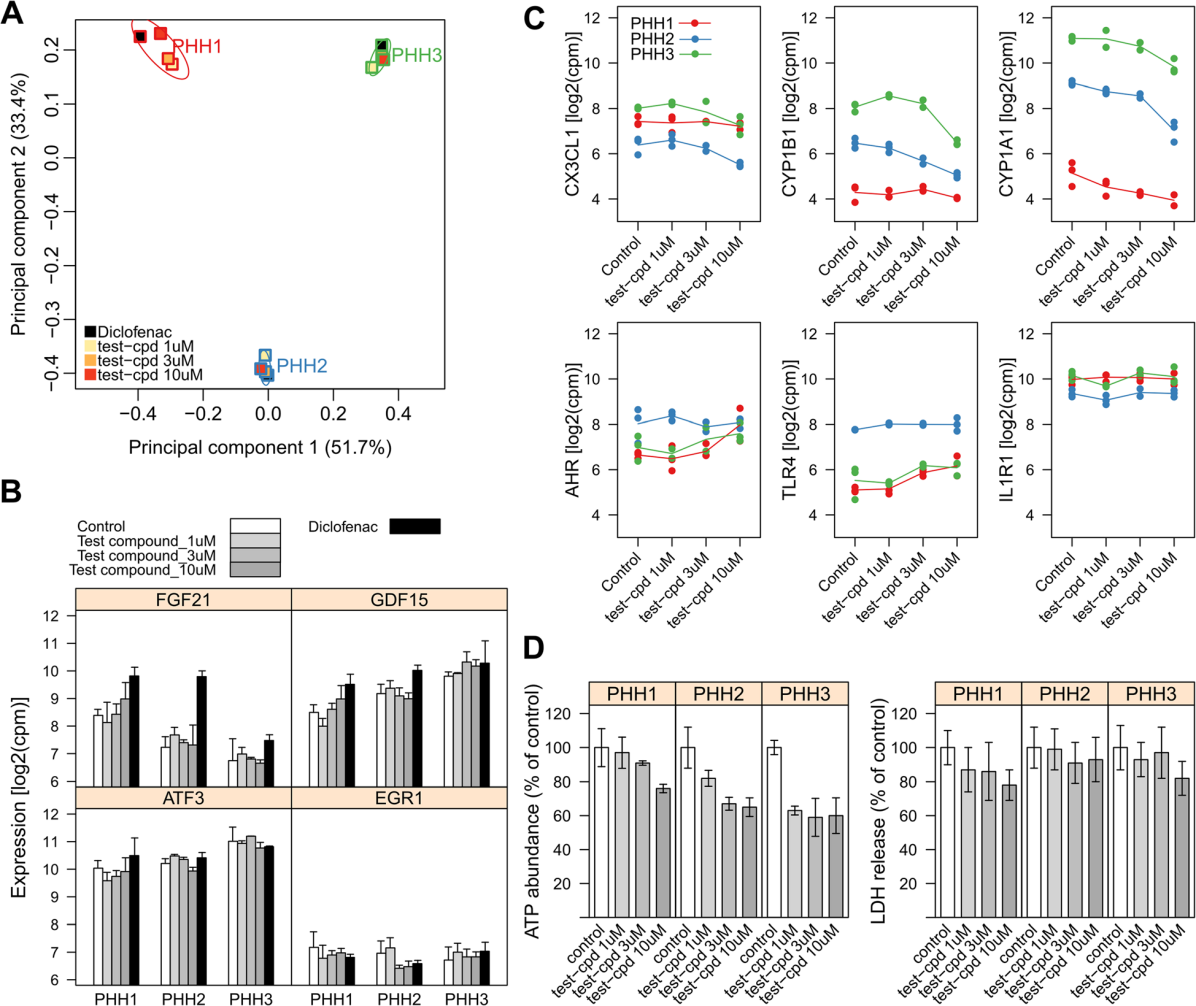
Molecular phenotyping of drug response in primary human hepatocytes. **(A)** Principal comment analysis of differential gene expression induced by Diclofenac and the test compound in three primary human hepatocyte cell lines. **(B)** Differential expression of *FGF21*, *GDF15*, *ATF3*, and *EGR1* induced by Diclofenac and test compound. The genes belong to an early induced stress-response network that is associated with cytotoxicity *in vitro* and *in vivo*. Average expression levels are expressed as log2-transformed copies per million reads (cpm). Error bars indicate standard deviation of two or three independent biological replicates. **(C)** Hypoxia signaling pathway reporter genes with dose-dependent gene expression profiles identified by AmpliSeq-RNA. Dots indicate mean expression of replicates. **(D)** Intracellular ATP abundance and LDH leakage in test compound treated cells measured by *in vitro* assays. Vertical bars indicate percentage (%) of average values of the control condition. Error bars indicate standard deviation of two or three independent biological replicates.

In addition, molecular phenotyping revealed transient activation of the Hedgehog (Smoothened) pathway (Figure 2C(d)), which can be attributed to the significant temporal regulation of key players *GLI1*, *SHH*, and *PTCH1* (the linear coefficient of time-dependent expression larger than zero, Benjamini-Hochberg adjusted p < 0.05, Figure 2D). Previous data established an essential role of the Hedgehog pathway in multiple processes involved in cardiomyocyte differentiation and heart morphogenesis in model species including mouse [29] and zebrafish [30]. Gene transfer with Sonic hedgehog (*Shh)* even repaired chronic myocardial ischemia in mice [31]. Our data suggest that the pathway is also involved in the *in vitro* differentiation of cardiomyocytes derived from induced human pluripotent stem cells. It raises the possibility of modeling and studying complex disease traits such as diabetic cardiomyopathy “in a dish” using the above mentioned iPSC system.

Cao *et al.* ([32]) and Wang *et al.* ([33]) provided data supporting expression and function of Toll-like receptors in embryonic and mesenchymal stem-cell differentiated cardiomyocyte models. Our data are in line with these observations, and further reveal that the TLR pathway is gradually activated up to day 60 of differentiation in iPSC-induced cardiomyocytes (Figure 2C(e)).

We observed the activation of interferon signaling pathway towards the end of the differentiation protocol (Figure 2C(f)). Previous studies showed that, at least in mouse, Stat1, a key transcription factor regulating interferon response genes, is an essential player in the innate response to viral diseases using an embryonic stem-cell model at day 12 or 13 [34]. Similarly, Stat3, another key transcription factor regulating the process, is essential for cardiomyocyte differentiation of mouse P19CL6 cells [35]. Molecular phenotyping data suggest that the interferon response pathway is induced at the late stage of differentiation together with other immune-response pathways. Its biological function in this context is not yet fully understood and remains to be studied.

Last but not least, we checked the matureness of the differentiated cardiomyocytes at day 60 at the pathway level. Several established cardiomyocyte-specific pathways, including the BMP-receptor mediated cardiomyocyte differentiation pathway (represented by atrial natriuretic peptides A and B *NPPA*/*NPPB*) [36], the extracellular matrix remodeling pathway (represented by *TIMP1* and *MMP1*) [37], the Neuregulin1/Erbb4 pathway (represented by *ERBB4*) [38], and muscle-specific Caveolin-mediated signaling pathways (represented by Caveolin 3 *CAV3*) [39], seemed to become most functional at day 20 and day 60.

For comparison, we performed differential gene expression analysis and GSVA analysis with the microarray data. We found that, while induction of some pathways and genes are equally well captured by microarray and molecular phenotyping (top panels of Figure 2C and E, Benjamini-Hochberg adjusted *p* < 0.05), several pathways and genes failed to be detected by the transcriptome-wide approach (bottom panels of Figure 2C and E, and Figure 2D, adjusted p > 0.05). There are two potential explanations: first, the background noise of microarray interferes with differential expression analysis of genes even they are lowly expressed, and many key regulators of pathways fall into this category of genes; second, the pathway reporter genes are enriched for key regulators and targets of signaling pathways, and therefore are potentially more informative about changes on the pathway level.

In summary, molecular phenotyping uncovered a highly coordinated program of pathway activation and inactivation during the differentiation of iPSC-induced cardiomyocytes. The novel approach establishes iPSC-induced cardiomyocytes as a suitable platform for disease characterization and drug discovery.

### Molecular phenotyping of adverse drug reactions

Following successful molecular phenotyping during differentiation of human iPS cells into cardiomyocytes, we applied the approach to understand the mechanistic basis of adverse effects. Our gene panel includes reporter genes for common toxicity mechanisms [40] and related pathways [41] and can potentially provide mechanistic clues to generic toxicity read-outs such as *in vitro* cytotoxicity (as measured *e.g.* by lactate dehydrogenase (LDH) release).

In contrast to common toxicogenomics approaches that monitor gene expression 24 or 48 hours after treatment, we wished to detect acute primary responses to drug exposure. Based on our previous findings such early responses can provide a consensus toxicity signature *in vitro* and *in vivo* [42]. Human primary hepatocyte assays are established in the field of predictive *in vitro* toxicity, and therefore we have chosen to analyze primary human hepatocytes (PHHs) from three donors in response to a proprietary test compound. As a positive control we have exposed the same cells to diclofenac, a non-steroidal anti-inflammatory drug known to induce idiosyncratic liver toxicity in the clinic [43]. The concentration of the test compound and diclofenac was on purpose chosen below the toxic concentration inducing LDH release to test if molecular phenotyping three hours after drug exposure already detects subtle effects hinting at toxicity at higher concentration and longer exposure.

First we performed principal component analysis for high level assessment of the experimental setup (Figure 3A). Surprisingly the inter-individual variation was found to dominate the clustering. However, a compound effect was still visible. Consistent with published literature [42], Diclofenac significantly up-regulated *FGF21* and *GDF15* as reporter genes of the early stress-response network (Benjamini-Hochberg adjusted p < 0.05). The response of this network to the test compound was significantly lower at all concentrations tested suggesting a different mode of action (Figure 3B). Transcriptional regulation of AT3 and EGR1, the other two genes in the early induced stress-response network, showed considerable variability between donors (Figure 3B, lower panels).

Strikingly, out of 154 pathways surveyed by the panel, only the hypoxia signaling network was significantly regulated (p < 0.001, one-sided Fisher’s exact test). Six pathway reporter genes *CX3CL1* [44], *CYP1B1*, *CYP1A1*, *AHR* [45], *TLR4* [46,47] and *IL1R1* [48] defining this cascade were all positively or negatively regulated in a dose-dependent manner (Benjamini-Hochberg adjusted *p* < 0.10, Figure 3C). The test compound induces specifically hypoxia signaling predominantly at 10 μM, suggesting the possibility that higher doses might cause safety concerns.

Hypoxia, the clinical depletion of oxygen supply, is associated with reduced metabolism resulting in ATP deficiency. This opened the possibility of functional validation of the hypothesis generated by molecular phenotyping. We exposed PHHs of the same donors to 1, 3 and 10 μM test compound for 24 hours and measured the intra-cellular ATP content and LDH release in parallel (Figure 3D). Consistent with our hypothesis, we observed a significant reduction of intracellular ATP in all donors with the expected inter-individual variation (coefficient of dose-dependent change less than zero, *p* < 0.01). The LDH levels were in contrast almost stable under all conditions, confirming the expected absence of cytotoxicity (Figure 3D).

This example shows that molecular phenotyping can distinguish pathways leading to adverse side effects of drugs thereby supporting preclinical drug safety assessment. The fact that test compound modulated a single pathway only out of 154 demonstrates specificity of the approach. Thus molecular phenotyping might develop into a valuable tool for early, mechanistic assessment of drug action and toxicity.

## Discussion and conclusions

In this study, we compiled a list of 917 human pathway reporter genes covering 154 signaling and metabolism networks. Accurate and rapid profiling of reporter genes by sequencing based RNA quantification allows derivation of a molecular phenotype in human cells. We identified temporal pathway activation patterns during differentiation of iPSC-derived cardiomyocytes, and inferred *in vitro* toxicity profiles of drugs in primary human hepatocytes. In both cases results of molecular phenotyping were validated by either literature information or parallel experiments.

Conventionally, reporter genes are selected for applications with a concrete, limited scope, for instance in our previous work on the ERBB-cell cycle signaling network [49] and on the white-to-brown conversion of white adipose [50]. In the current study, we attempt to capture a wide range of biological pathways by collecting reporter genes using an integrative approach. We demonstrate that a molecular phenotypic screening approach can be used to monitor pathway activation patterns in different cellular systems using the latest AmpliSeq-RNA technology. The workflow is fast (~4 h processing time) and can be adapted for screening purposes once the costs per array decrease. Given the high sensitivity, wide coverage of pathways, and the high throughput, we anticipate the new approach will empower more phenotypic screening in a wide range of biological applications.

The pathway reporter gene panel can be expanded with application-specific plug-ins following the *LEGO* principle. In a vaccination study, for instance, we have added an immune panel consisting of ~600 genes specific to innate and adaptive immune pathways, to enhance the resolution of the standard panel. The two panels reported consistent data on pathway activities, and a fine-grained snapshot of immune-relevant biological processes within one experiment (Lenz *et al.*, manuscript submitted). Panels focusing on other specific biological processes, such as toxicity, apoptosis, proliferation, drug metabolism, *etc.*, can be designed and used jointly with the core panel to extract detailed information from the biological system of interest.

Using amplicon-based sequencing technology circumvents some computational challenges associated with microarray or conventional RNAseq, and much of the workflow has been automated so that users can work with a matrix of read counts a few hours after the sequencing is finished. In the present study, several exploratory data analysis approaches were used to compare effects of treatments and to extract pathway-level information, including PCA, GSVA, differential gene expression and functional enrichment analysis based on Fisher’s exact test. We believe that data interpretation of molecular phenotypic screenings can both benefit from the abundance of tools that have been developed predominantly for transcriptome-wide expression data [51-54] and standardized by using specialized tools that are being developed (Zhang *et al.*, manuscript in preparation).

Besides surveying pathway reporter genes, there are also other alternatives to the holistic approach of transcriptome expression profiling. For example, following a data-driven approach, Peck *et al.* [55] selected 1,000 genes (‘Landmark 1000’) that maximally conserve between-sample variability, and reconstructed transcriptome expression profiles from the 1000 genes using statistical models trained by public microarray data. More recently, Li *et al.* [56] reported a next-generation sequencing based high-throughput screening platform (HTS^2^) to monitor compound-induced gene expression changes that are associated with a given pathway (e.g. the androgen signaling pathway). These studies demonstrated good performance of signature-based disease classification and identified novel compounds with desired effects, underlining the power of reductionist approaches. Instead of focusing on expression profile reconstruction or on the activation pattern of single pathways, the molecular phenotyping approach aims to provide a comprehensive overview of major human signaling and metabolic pathways that are currently known. We believe that molecular phenotyping is a valuable tool for translational research and drug discovery.

## Methods

### Pathway reporter genes selection

Text mining of MEDLINE-index abstracts was performed using Linguamatics I2E software. The gene list of QIAGEN qRT-PCR panels and of Bio-Rad Tier 1, 2, and 3 panels was downloaded from the respective website and processed using python scripts. Data was retrieved from respective sources in November 2012.

PageRank algorithm was implemented in the *igraph* package [57] and default parameters were used. The SICORE algorithm was run with a large number of repeats (N = 1,000,000). When p < 0.05 was used as significance level, 675 pairs of genes were detected to share significantly more common pathways than the null model. In each pair the gene with the lower PageRank centrality was removed from the list. For each co-expression cluster, which is a collection of genes that are strongly co-expressed in published datasets (correlation coefficient >0.90 reported by COXPRESSdb), only the one with the highest PageRank centrality was retained and the others were removed.

### Functional enrichment analysis

Functional enrichment analysis was performed using one-sided Fisher’s exact test with GO/BP terms if not otherwise stated. Only annotations with experimental evidence codes were used.

Reporter genes’ relevance for human diseases was determined by comparing distribution of their associated diseases with randomly selected gene sets of the same size (repeated N = 1,000 times).

### Biological validation of the gene selection

#### Differentiation of pluripotent stem cells into cardiomyocytes

Experimental design and protocols have been described in previous publications [22,28]. The GSVA algorithm was run with the ‘NGS’ mode and otherwise default parameters. Gene-level differential expression of reporter genes was identified with the *edgeR* package [58]. Microarray data were analyzed with the *limma* package [59]. In both cases, a linear model was set up and a polynomial contrast including the linear and the quadratic term was used to test differential expression. Significant differential expressed genes were called using the following threshold if not otherwise stated: absolute log fold-change > =1 and Benjamini-Hochberg adjusted *p* < 0.05.

### In vitro toxicity evaluation in primary hepatocytes

Cryopreserved primary human hepatocytes were purchased form BioreclamationIVT (Brussels, Belgium), who obtains and distributes consented human material from a network of institutional review board (IRB) approved collection sites under adherence to effective ethical and regulatory guidelines. The cells were thawed according to manufacturer’s instructions, and cultured at a density of 35’000 cells/well in 96-well BD BioCoat Collagen 1 plates (Becton Dickinson, Bedford, MA). Cells were exposed to test compounds or vehicle (0.1% DMSO) after over-night pre-culture in William’s Medium E (Sigma-Aldrich, Buchs, Switzerland) supplemented with Penicillin/Streptomycin (Life Technologies, Zug, Switzerland).

Cell were harvested 3 h after test compound addition by removing the culture medium. Cell lysates in 50 μl RLT buffer (QIAGEN, Hombrechtikon, Switzerland) were immediately frozen and stored at −80°C.

Intracelluar ATP content was assessed 24 h after test compound addition using the CellTitre-Glo assay (Promega, Dübendorf, Switzerland).

Cell integrity was assessed by the release of the intracellular enzyme lactate dehydrogenase (LDH) into the cell culture supernatant 24 h after test compound addition using the Cytotoxicity Detection Kit (Roche Diagnostics, Rotkreuz, Switzerland).

## Additional files

Additional file 1: Table S1. List of reporter genes and pathways. Additional file 2: Table S2. AmpliSeq identifiers and primer pairs of reporter genes. Additional file 3:
**Heatmap of pathway activity as in Figure** 2 **B, with pathway names.**

## Abbreviations

qRT-PCR: Quantitative reverse transcription polymerase chain reaction
GO/ BP: Gene Ontology/ Biological Process
iPSC: Induced pluripotent stem-cell
PCA: Principal component analysis
GSVA: Gene set variation analysis
PHH: Primary human hepatocyte
