# Erratum to: Pathway reporter genes define molecular phenotypes of human cells

**DOI:** 10.1186/s12864-017-3730-6

**Published:** 2017-05-02

**Authors:** Jitao David Zhang, Erich Küng, Franziska Boess, Ulrich Certa, Martin Ebeling

**Affiliations:** Pharmaceutical Research and Early Development, Pharmaceutical Sciences, Roche Innovation Center Basel, F. Hoffmann-La Roche AG, Grenzacherstrasse 124, 4070 Basel, Switzerland

## Erratum

After the publication of this work [1] it was noticed that the 3 additional files do not match the corresponding captions. Please see the correct Additional files 1, 2 and 3 below.

## Additional files

Additional file 1: Table S1. List of reporter genes and pathways. (XLSX 398 kb)

Additional file 2: Table S2. AmpliSeq identifiers and primer pairs of reporter genes. (XLSX 84 kb)

Additional file 3: Heatmap of pathway activity as in Figure 2B, with pathway names. (PNG 294 kb)
