# Supplementary figures and images for: Erratum to: Pathway reporter genes define molecular phenotypes of human cells
Source: BMC Genomics. 2017 May 2;18:337. doi: 10.1186/s12864-017-3730-6 (PMC5412029; doi:10.1186/s12864-017-3730-6)

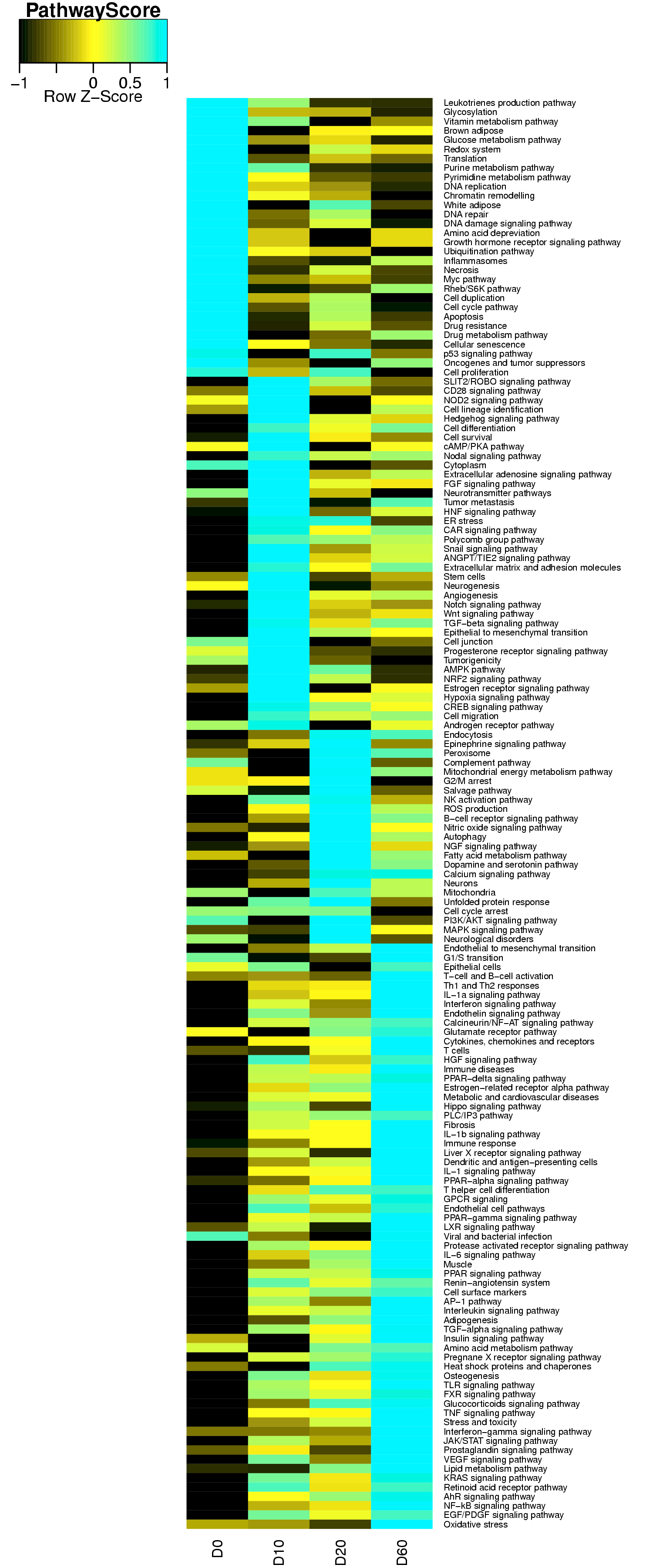

Supplement: Supplementary file 3 — Heatmap of pathway activity as in Figure 2B, with pathway names. (PNG 294 kb) [file 12864_2017_3730_MOESM3_ESM.png]
